# Use of Antihypertensives, Blood Pressure, and Estimated Risk of Dementia in Late Life: An Individual Participant Data Meta-Analysis

**DOI:** 10.1001/jamanetworkopen.2023.33353

**Published:** 2023-09-12

**Authors:** Matthew J. Lennon, Ben Chun Pan Lam, Darren M. Lipnicki, John D. Crawford, Ruth Peters, Aletta E. Schutte, Henry Brodaty, Anbupalam Thalamuthu, Therese Rydberg-Sterner, Jenna Najar, Ingmar Skoog, Steffi G. Riedel-Heller, Susanne Röhr, Alexander Pabst, Antonio Lobo, Concepción De-la-Cámara, Elena Lobo, Toyin Bello, Oye Gureje, Akin Ojagbemi, Richard B. Lipton, Mindy J. Katz, Carol A. Derby, Ki Woong Kim, Ji Won Han, Dae Jong Oh, Elena Rolandi, Annalisa Davin, Michele Rossi, Nikolaos Scarmeas, Mary Yannakoulia, Themis Dardiotis, Hugh C. Hendrie, Sujuan Gao, Isabelle Carrière, Karen Ritchie, Kaarin J. Anstey, Nicolas Cherbuin, Shifu Xiao, Ling Yue, Wei Li, Maëlenn M. Guerchet, Pierre-Marie Preux, Victor Aboyans, Mary N. Haan, Allison E. Aiello, Tze Pin Ng, Ma Shwe Zin Nyunt, Qi Gao, Marcia Scazufca, Perminder S. S. Sachdev

**Affiliations:** 1 Faculty of Medicine, University of New South Wales, Sydney, Australia; 2 Centre for Healthy Brain Aging, Discipline of Psychiatry & Mental Health, School of Clinical Medicine, University of New South Wales, Sydney, Australia; 3 School of Psychology and Public Health, La Trobe University, Melbourne, Australia; 4 The George Institute for Global Health, Sydney, Australia; 5 School of Biomedical Sciences, University of New South Wales, Sydney, Australia; 6 School of Public Health, Imperial College London, London, United Kingdom; 7 School of Population Health, University of New South Wales, Sydney, Australia; 8 Eastern Suburbs Older Persons’ Mental Health Service, Sydney, Australia; 9 Neuropsychiatric Epidemiology Unit, Department of Psychiatry and Neurochemistry, Institute of Neuroscience and Physiology, The Sahlgrenska Academy, Centre for Ageing and Health at the University of Gothenburg, Gothenburg, Sweden; 10 Aging Research Center, Department of Neurobiology, Care Sciences and Society, Karolinska Institutet and Stockholm University, Stockholm, Sweden; 11 Psychiatry, Cognition and Old Age Psychiatry Clinic, Region Västra Götaland, Sahlgrenska University Hospital, Gothenburg, Sweden; 12 Institute of Social Medicine, Occupational Health and Public Health, Medical Faculty, University of Leipzig, Leipzig, Germany; 13 School of Psychology, Manawatu Campus, Massey University, Palmerston North, New Zealand; 14 Global Brain Health Institute, Trinity College Dublin, Dublin, Ireland; 15 Department of Medicine and Psychiatry, Universidad de Zaragoza, Zaragoza, Spain; 16 Instituto de Investigación Sanitaria Aragón, Zaragoza, Spain; 17 Centro de Investigación Biomédica en Red de Salud Mental, Madrid, Spain; 18 World Health Organization Collaborating Centre for Research and Training in Mental Health, Neuroscience, and Substance Abuse, Department of Psychiatry, College of Medicine, University of Ibadan, Ibadan, Nigeria; 19 Department of Neurology, Albert Einstein College of Medicine, Bronx, New York; 20 Department of Epidemiology and Population Health, Albert Einstein College of Medicine, Bronx, New York; 21 Department of Neuropsychiatry, Seoul National University Bundang Hospital, Seongnam, Korea; 22 Department of Psychiatry, Seoul National University College of Medicine, Seoul, Korea; 23 Department of Brain and Cognitive Sciences, Seoul National University College of Natural Sciences, Seoul, Korea; 24 Workplace Mental Health Institute, Kangbuk Samsung Hospital, Sungkyunkwan University School of Medicine, Seoul, Korea; 25 Golgi Cenci Foundation, Abbiategrasso, Italy; 26 Department of Brain and Behavioural Sciences, University of Pavia, Pavia, Italy; 27 First Department of Neurology, Aiginition Hospital, National and Kapodistrian University of Athens, Athens, Greece; 28 Department of Neurology, Columbia University, New York, New York; 29 Department of Nutrition and Dietetics, School of Health Sciences and Education, Harokopio University, Athens, Greece; 30 Department of Neurology, University Hospital of Larissa, Larissa, Greece; 31 Faculty of Medicine, School of Health Sciences, University of Thessaly, Larissa, Greece; 32 Department of Psychiatry, Indiana University School of Medicine, Indianapolis; 33 Indiana Alzheimer Disease Research Center, Indiana Alzheimer Disease Research Center, Indianapolis; 34 Department of Biostatistics and Health Data Science, Indiana University School of Medicine, Indianapolis; 35 Institut for Neurosciences of Montpellier, University Montpellier, National Institute for Health and Medical Research, Montpellier, France; 36 Institut du Cerveau Trocadéro, Paris, France; 37 University of New South Wales, School of Psychology, Sydney, Australia; 38 Ageing Futures Institute, University of New South Wales, Sydney, Australia; 39 Neuroscience Research Australia, Sydney, Australia; 40 National Centre for Epidemiology and Population Health, Australian National University, Canberra, Australia; 41 Department of Geriatric Psychiatry, Shanghai Mental Health Center, Shanghai Jiao Tong University School of Medicine, Shanghai, China; 42 Alzheimer’s Disease and Related Disorders Center, Shanghai Jiao Tong University, Shanghai, China; 43 National Institute for Health and Medical Research U1094, Institut de Recherche pour le Developpement UMR270, Epidemiology of Chronic Diseases in Tropical Zone, Institute of Epidemiology and Tropical Neurology, OmegaHealth, University Limoges, Centre Hospitalier et Universitaire Limoges, Limoges, France; 44 Department of Cardiology, Dupuytren 2 University Hospital, Limoges, France; 45 School of Medicine, University of California, San Francisco; 46 Robert N. Butler Columbia Aging Center, Department of Epidemiology, Mailman School of Public Health, Columbia University, New York, New York; 47 Department of Psychological Medicine, Yong Loo Lin School of Medicine, National University of Singapore, Singapore, Singapore; 48 Geriatric Education and Research Institute, Ministry of Health, Singapore, Singapore; 49 Departamento de Psiquiatria, Faculdade de Medicina, Universidade de Sao Paulo, Sao Paulo, Brazil; 50 Neuropsychiatric Institute, Prince of Wales Hospital, Sydney, Australia

## Abstract

**Importance:**

The utility of antihypertensives and ideal blood pressure (BP) for dementia prevention in late life remains unclear and highly contested.

**Objectives:**

To assess the associations of hypertension history, antihypertensive use, and baseline measured BP in late life (age >60 years) with dementia and the moderating factors of age, sex, and racial group.

**Data Source and Study Selection:**

Longitudinal, population-based studies of aging participating in the Cohort Studies of Memory in an International Consortium (COSMIC) group were included. Participants were individuals without dementia at baseline aged 60 to 110 years and were based in 15 different countries (US, Brazil, Australia, China, Korea, Singapore, Central African Republic, Republic of Congo, Nigeria, Germany, Spain, Italy, France, Sweden, and Greece).

**Data Extraction and Synthesis:**

Participants were grouped in 3 categories based on previous diagnosis of hypertension and baseline antihypertensive use: healthy controls, treated hypertension, and untreated hypertension. Baseline systolic BP (SBP) and diastolic BP (DBP) were treated as continuous variables. Reporting followed the Preferred Reporting Items for Systematic Review and Meta-Analyses of Individual Participant Data reporting guidelines.

**Main Outcomes and Measures:**

The key outcome was all-cause dementia. Mixed-effects Cox proportional hazards models were used to assess the associations between the exposures and the key outcome variable. The association between dementia and baseline BP was modeled using nonlinear natural splines. The main analysis was a partially adjusted Cox proportional hazards model controlling for age, age squared, sex, education, racial group, and a random effect for study. Sensitivity analyses included a fully adjusted analysis, a restricted analysis of those individuals with more than 5 years of follow-up data, and models examining the moderating factors of age, sex, and racial group.

**Results:**

The analysis included 17 studies with 34 519 community dwelling older adults (20 160 [58.4%] female) with a mean (SD) age of 72.5 (7.5) years and a mean (SD) follow-up of 4.3 (4.3) years. In the main, partially adjusted analysis including 14 studies, individuals with untreated hypertension had a 42% increased risk of dementia compared with healthy controls (hazard ratio [HR], 1.42; 95% CI 1.15-1.76; *P* = .001) and 26% increased risk compared with individuals with treated hypertension (HR, 1.26; 95% CI, 1.03-1.53; *P* = .02). Individuals with treated hypertension had no significant increased dementia risk compared with healthy controls (HR, 1.13; 95% CI, 0.99-1.28; *P* = .07). The association of antihypertensive use or hypertension status with dementia did not vary with baseline BP. There was no significant association of baseline SBP or DBP with dementia risk in any of the analyses. There were no significant interactions with age, sex, or racial group for any of the analyses.

**Conclusions and Relevance:**

This individual patient data meta-analysis of longitudinal cohort studies found that antihypertensive use was associated with decreased dementia risk compared with individuals with untreated hypertension through all ages in late life. Individuals with treated hypertension had no increased risk of dementia compared with healthy controls.

## Key Points

**Question:**

Are blood pressure (BP) and treatment for hypertension in late life associated with dementia risk?

**Findings:**

In this meta-analysis including individual participant data from 34 519 community dwelling older adults in 17 studies, untreated hypertension was associated with a greater risk of dementia compared with treated hypertension, and this association was not modified by age. Participants with treated hypertension had no greater dementia risk compared with healthy controls, and baseline BP did not moderate the reduced dementia risk in participants with treated hypertension.

**Meaning:**

The findings indicate that ongoing antihypertensive therapy throughout late life is an important part of dementia prevention.

## Teaser

This meta-analysis using individual participant data assesses the associations of hypertension history, antihypertensive use, and baseline blood pressure with dementia risk in adults aged older than 60 years.

## Introduction

Hypertension is the most prevalent risk factor for dementia, affecting more than 1 billion people worldwide.^1^ Midlife hypertension is associated with an approximately 60% increased risk of all-cause dementia^2^ and an approximately 25% increased risk of Alzheimer dementia.^3^ However, in late life, this association was not consistently observed, and most studies have found either no association or that higher systolic blood pressure (SBP) or diastolic blood pressure (DBP) was associated with lower risk of dementia.^4^

A recent individual participant data (IPD) meta-analysis^5^ including 17 286 participants (age range, 65-95 years) found that higher BP may have a protective association against dementia. The meta-analysis by van Dalen et al^5^ found a negative, approximately linear association, indicating that higher SBP was associated with lower risk of dementia and the low point of risk was at an SBP of approximately 185 mm Hg, although this result was modified by age.^5^ Other studies of late-life BP have found U-shaped associations between BP and dementia risk, but estimates of the lowest-risk BPs vary widely.^6^ Aside from differential associations with changing age, studies have also indicated that the association of BP with dementia risk is moderated by sex^7,8,9,10,11,12,13,14^ and racial^15,16^ grouping. A systematic review^7^ found that for women, higher midlife SBP, but not late-life SBP, was associated with greater risk of dementia compared with men in 6 of 7 studies. A large study^17^ using US Medicare data examining late-life (age >65 years) adults, including 320 720 Black adults and 3 121 553 White adults, found that hypertension was associated with a greater AD risk in Black populations than in White populations. Several large studies^15,16^ examining cognitive outcomes have corroborated this finding.

While population-based studies of older persons (age >60 years) have regularly found higher BPs to be associated with lower dementia risk, clinical trials of antihypertensives indicate that lower BP targets produce the best cognitive outcomes.^8,18,19^ A 2022 IPD meta-analysis^20^ of randomized clinical trials including 28 008 participants found an adjusted odds ratio of 0.87 favoring treatment of hypertension for dementia risk reduction. There were no differences between male and female outcomes, although age significantly moderated the association, with considerably less benefit found in participants aged 80 years or older compared with those aged 61 to 70 years.

However, a key challenge with randomized clinical trials is their limited generalizability.^21,22^ They have strict inclusion criteria that exclude many participants, particularly in highly comorbid, elderly populations, and they are run almost exclusively in developed nations. Thus, there is a need for inclusive longitudinal studies that incorporate diverse populations to inform guidelines. In this study, we performed an IPD meta-analysis on a harmonized data set of 17 longitudinal studies^23,24,25,26,27,28,29,30,31,32,33,34,35,36,37,38,39^ from across the world to delineate the precise associations of BP and antihypertensive use with the risk of progressing to dementia, as well as to understand the differential associations of BP with dementia in various age, sex, and racial groups.

## Methods

For this IPD meta-analysis, ethics approval was granted by the University of New South Wales Human Research Ethics Committee. Each contributing study had individual ethics approval from their respective institutions, and participants in each study provided informed consent (eTable 1 in Supplement 1). This study is presented according to the Preferred Reporting Items for Systematic Reviews and Meta-analyses of Individuals Participant Data (PRISMA-IPD) reporting guidelines.^40^

### Contributing Studies

Our analyses included 17 studies^23,24,25,26,27,28,29,30,31,32,33,34,35,36,37,38,39^ and 34 519 participants from the Cohort Studies of Memory in an International Consortium (COSMIC), which has been previously described in detail.^41,42,43,44^ All studies were longitudinal, population-based studies of aging that included measures of cognition and dementia status (Table 1). The cohorts were from 16 countries: US,^27,31,35^ Brazil,^37^ Australia,^34,38^ China,^29^ Korea,^25^ Singapore,^36^ Central African Republic,^26^ Republic of Congo,^26^ Nigeria,^27,28^ Germany,^33^ Spain,^39^ Italy,^24^ France,^23^ Sweden,^32^ and Greece.^30^ They had various assessment schedules (2-16 waves) and follow-up durations (2-15 years) (Table 1). Further descriptions of the studies, including covariates and harmonization protocols, are detailed in eTables 2 through 8 in Supplement 1.

**Table 1.** Summary of Demographics at Baseline and Dementia Rates of the 17 Studies Included in Cohort Studies of Memory in an International Consortium After Exclusions

Study	Study name	Main racial group (country)	Age, mean (SD), y	Male sex, %	Education, mean (SD), y	Maximum waves, No.	Follow-up, y		Blood pressure, Mean (SD), mm Hg		Hypertension status, No. (%)	Dementia, No. (%)	Time to dementia diagnosis, mean (SD), y
							Maximum, No.	Mean (SD)	Systolic	Diastolic			
Xiao et al,^29^ 2016	Chinese Longitudinal Aging Study	Asian (China)	71.1 (7.8)	45.4	7.7 (5.3)	3	7.2	1 (1.5)	129.5 (15.2)	77.9 (8.7)	HC: 1318 (51.5); UCH: 11 (0.4); TH: 1108 (43.3); UTH: 121 (4.7)	60 (2.8)	0.5 (0.1)
Guerchet et al,^26^ 2014	Epidemiology of dementia in Central Africa	Black (Central African Republic and Republic of Congo)	73.1 (6.6)	41.1	2.0 (3.7)	4	2.9	0.8 (1.1)	142.1 (26.7)	80.9 (13.4)	HC: 0; UCH: 0; TH: 175 (38); UTH: 286 (62)	19 (5.9)	1.5 (0.8)
Dardiotis et al,^30^ 2014	The Hellenic Longitudinal Investigation of Aging and Diet	White (Greece)	72.8 (5.5)	40.1	8.1 (5)	2	7.3	1.7 (1.7)	131.7 (17.7)	77.4 (9.9)	HC: 480 (26); UCH: 165 (8.9); TH: 1114 (60.3); UTH: 89 (4.8)	62 (3.3)	1.6 (0.4)
Hendrie et al,^27^ 2001	Indianapolis-Ibadan Study	Black (Nigeria)	73.6 (5.9)	27.8	1.2 (3.2)	7	17.7	5.7 (4.7)	155.3 (32.7)	85.9 (16)	NA	216 (13.1)	5.6 (3.9)
Hendrie et al,^27^ 2001	Indianapolis-Ibadan Study	Black (US)	75.7 (6)	33.4	11.0 (3.1)	7	17.4	4.9 (4.3)	146.9 (22.2)	80.3 (11.8)	NA	262 (18)	5.2 (3.8)
Katz et al,^31^ 2011	Einstein Aging Study	Black and White (US)	78.1 (5.3)	38.2	13.2 (3.6)	16	19.6	2.7 (3.4)	134.1 (15.9)	77.4 (8.5)	HC: 591 (29.7); UCH: 207 (10.4); TH: 1019 (51.2); UTH: 173 (8.7)	153 (7.4)	3.8 (3.3)
Ritchie et al,^23^ 2010	Etude Santé Psychologique Prévalence Risques et Traitement	White (France)	73.1 (5.5)	41.6	10.2 (3.8)	4	9	9.3 (5.6)	140.9 (17.4)	79.7 (9.9)	HC: 1191 (54.8); UCH: 182 (8.4); TH: 766 (35.2); UTH: 36 (1.7)	209 (9.6)	6.9 (4.5)
Rydberg Sterner et al,^32^ 2019	Gothenburg H70 Birth Cohort Studies	White (Sweden)	73.3 (4.9)	28.9	9.7 (3.7)	3	10.7	5.9 (4.1)	155.6 (21.8)	84.5 (11.3)	HC: 483 (57.6); UCH: 86 (10.3); TH: 239 (28.5); UTH: 30 (3.6)	124 (15.8)	5.9 (2.8)
Guaita et al,^24^ 2013	Brain Ageing in Abbiategrasso	White (Italy)	72.2 (1.3)	46.0	6.8 (3.3)	2	3.3	3.4 (1.4)	141.7 (17.5)	78.9 (8.4)	HC: 444 (35); UCH: 63 (5); TH: 729 (57.4); UTH: 34 (2.7)	59 (4.6)	2.4 (1)
Han et al,^25^ 2018	Korean Longitudinal Study on Cognitive Aging and Dementia	Asian, (Korea)	69.9 (6.6)	43.6	8.2 (5.3)	4	7.1	3.9 (2.4)	126.2 (14.8)	77.9 (9.2)	HC: 1137 (29.5); UCH: 161 (4.2); TH: 2233 (58); UTH: 320 (8.3)	226 (3.7)	2.6 (1.5)
Reidel-Heller et al,^33^ 2001	Leipzig Longitudinal Study of the Aged	White (Germany)	81.5 (4.9)	25.9	11.9 (1.7)	7	16	4.7 (3.4)	158.6 (24.3)	86.1 (16.2)	NA	229 (23.2)	3.6 (2.7)
Anstey et al,^34^ 2012	Personality and Total Health Through Life Study	White (Australia)	62.5 (1.5)	51.5	13.7 (2.8)	4	13.9	9.7 (4.5)	139.8 (19.5)	83 (10.7)	HC: 1456 (58.7); UCH: 0; TH: 822 (33.1); UTH: 202 (8.1)	80 (3.2)	8.9 (2.6)
Haan et al,^35^ 2003	Sacramento Area Latino Study on Aging	Mexican origin (US)	70.4 (6.8)	41.6	7.3 (5.3)	7	9.4	5.5 (3.2)	138.5 (19.3)	75.9 (10.6)	HC: 552 (32.5); UCH: 0; TH: 719 (42.3); UTH: 429 (25.2)	116 (6.8)	1 (0)
Feng et al,^36^ 2010	Singapore Longitudinal Aging Study	Asian (Singapore)	68 (5.6)	38.9	6.2 (4.6)	3	4.6	2 (1.7)	135 (17.2)	81.5 (9.1)	HC: 999 (50.7); UCH: 5 (0.3); TH: 883 (44.8); UTH: 82 (4.2)	13 (3.3)	3.1 (0.7)
Scazufca et al,^37^ 2008	São Paulo Aging & Health Study	Multiple (Brazil)	72 (6.1)	39.5	2.5 (3)	2	4.1	1.8 (0.9)	145.7 (25.7)	86 (13.6)	HC: 378 (19.6); UCH: 0; TH: 1142 (59.3); UTH: 407 (21.1)	38 (2.1)	2 (0)
Sachdev et al,^38^ 2010	Sydney Memory and Aging Study	White (Australia)	78.8 (4.8)	44.8	11.6 (3.5)	4	6.8	4.6 (2.1)	144.6 (20.8)	81.8 (10.8)	HC: 306 (29.6); UCH: 98 (9.5); TH: 570 (55.2); UTH: 59 (5.7)	178 (17.2)	4.8 (2.2)
Lobo et al,^39^ 2005	Zaragoza Dementia Depression Project	White (Spain)	73.9 (9.3)	42.9	7.1 (3.8)	3	6.7	2.9 (2.1)	141.3 (18.7)	79.1 (11.2)	HC: 123 (6.9); UCH: 0; TH: 1410 (79.3); UTH: 246 (13.8)	137 (3.1)	2.2 (1.2)
Hall et al,^28^ 1998	Ibadan Study of Aging (ISA)	Black (Nigeria)	78.2 (8.8)	49.9	4.1 (5.2)	4	6	2 (0)	156.5 (26.6)	85.1 (13.6)	NA	51 (2.8)	0.7 (0.4)
Total	NA	NA	72.5 (7.7)	41.6	8.2 (5.4)			4.3 (4.3)	138.7 (21.5)	80.2 (11.3)	HC: 10 402 (35.5); UCH: 1293 (4.4); TH: 14 759 (50.3); UTH: 2881 (9.8)	2232 (6.5)	4.1 (3.5)

Abbreviations: HC, health controls; NA, not applicable; TH, treated with antihypertensives; UCH, uncertain hypertension status; UTH, untreated hypertension.

### Measures of BP, Hypertension History, Antihypertensive Use, and Covariates

All studies had data on self-reported diagnosis of hypertension, and 14 studies^23,24,25,26,29,30,31,32,34,35,36,37,38,39^ included antihypertensive use at baseline. All studies had between 1 and 3 direct measures of BP at baseline, obtained with participants seated. Details on the methods of BP measurement in each study are in eTable 5 in Supplement 1. For studies with more than 1 measure of BP at each wave, the means of those measures (seated only) were taken. Participants with BP 3 SDs from the overall mean across studies were excluded as outliers (ie, SBP: <73.1 mm Hg or >204.1 mm Hg; DBP: <45.1 mm Hg or >114.4 mm Hg). Numbers and percentages of excluded participants can be found in eTable 2 in Supplement 1. Covariates included age, sex, education level, race, body mass index, diabetes status, hypercholesterolemia, and smoking status. Race was self-reported in the individual studies and categorized as Asian, Black, White, and other (encompassing a range of different groups that did not fit within the other categories, eg, American Indian or First Nations Australian). Race was included in the analyses because previous studies have found that hypertension is differentially associated with dementia risk in different racial groups. The categorization and harmonization processes are described in eTables 5 through 7 in Supplement 1.

### Dementia Outcome

The key outcome for this study was all-cause dementia. Most studies diagnosed dementia using *Diagnostic and Statistical Manual of Mental Disorders* (Fourth Edition) (*DSM-IV*) criteria, although some used *Diagnostic and Statistical Manual of Mental Disorders* (Third Edition Revised) (*DSM III-R*) criteria (eTable 8 in Supplement 1). Dementia onset was assigned a date midway between the assessment date when dementia was first diagnosed and the previous assessment date. Three studies^23,24,25^ provided dementia diagnosis dates that occurred in medical visits outside of the formal study, and these dates were treated as dates of dementia onset. Participants with dementia at baseline, as defined by each study, were excluded from our analyses.

### Statistical Analysis

For each of our analyses, a 1-step IPD approach was used, ie, models were run for all participants in a combined data set with a random-effect term for study, rather than running the models in individual studies and pooling them using a random-effects meta-analysis. This approach was used because our meta-analyses included small studies with low event rates, where interrogation of interaction effects has reduced power in 2-step approaches.^45^ Hypertension history was examined as a dichotomous variable, but its association was considerably modified by treatment status (eTable 9 in Supplement 1); thus, our main analysis focused on a categorical variable for hypertension based on both reported hypertension history and antihypertensive use. There were 4 possible groups defined by this variable: no hypertension history while not using an antihypertensive at baseline, classified as healthy control participants; no hypertension history while using an antihypertensive at baseline, classified as uncertain hypertension; reporting hypertension history while using an antihypertensive at baseline, classified as treated hypertension; and reporting hypertension history while not using an antihypertensive at baseline, classified as untreated hypertension. Given that individuals with no reported hypertension history who were using an antihypertensive had an unclear hypertension history, they were excluded from this part of the analysis (1296 participants [4.2%]) (eMethods in Supplement 1). These groupings formed a key part of our analysis, and as such, a between-group comparison of characteristics was performed (eTable 4 in Supplement 1). Diagnosis of hypertension requires at least 2 BP measures taken at least 1 month apart^46^; hence, the single measure of baseline BP was not included in our definition of hypertension and antihypertensive status.

Regarding continuous measures of BP, SBP and DBP were centered on the overall mean (ie, SBP: 140 mm Hg; DBP: 80 mm Hg) and divided by 5 (ie, measured in units of 5 mm Hg) to ensure effect sizes would be comparable with other covariates. Previous studies have shown that BP has a U-shaped or parabolic association with dementia.^5,6^ These potential nonlinear associations were examined using natural splines terms for SBP and DBP, with 2 to 4 degrees of freedom according to optimal fit (using Akaike Information Criteria and Bayesian Information Criteria). Similarly, studies have found that risk of dementia increases parabolically rather than linearly with age.^47^ Thus, age was centered on the overall mean (ie, 73 years), and both linear and quadratic age terms (age and age squared) were included in all analyses. Participants younger than 60 years were excluded from the study as they were not considered to be in late life.

Mixed-effects Cox proportional hazards survival models were used to assess the association between various measures and progression to dementia. The first analysis examined dementia risk associated with hypertension and antihypertensive status. The second assessed the associations of baseline SBP and DBP with dementia using natural splines to model the association. Initially, models including continuous BP parameters and hypertension and antihypertensive status, as well as their interaction terms, were examined, but these terms were not included in later models based on poor model fit, number of excluded participants, and lack of interaction significance.

The main analysis included as covariates only age, age squared, sex, education, racial group, and a random intercept term for study. This parsimonious, partially adjusted model was adopted as the main analysis to minimize the exclusion of studies, particularly from lower socioeconomic regions, which were more frequently lacking the other covariates. Further analyses were performed to assess the robustness of our results. First, a fully adjusted analysis was run, controlling additionally for body mass index, hypercholesterolemia, diabetes, and smoking status, using only participants and studies in which these variables were available. Second, a restricted analysis excluding individuals with less than 5 years of follow-up was run. This step was taken because dementia develops over many years; thus, occurrence of dementia within several years of baseline is likely caused by factors considerably prior to the study baseline. Third, to assess individual contributions of each of the studies and heterogeneity between studies, the main model was run within each individual study and results were examined for heterogeneity or outliers. Finally, to assess possible moderating factors of age, sex, and racial group, interactions with these variables were included in separate models. Further details of the interaction analyses are included in the eMethods in Supplement 1.

The Sydney COSMIC team generated the harmonized data set and ran the mixed effects Cox regressions using the coxme^48^ and splines packages in R statistical software version 4.0.3. *P* values were 2-sided, and *P* = .05 was considered significant. Data were analyzed from January to April 2023.

## Results

### Participant Characteristics

Of 34 519 participants in 17 studies^23,24,25,26,27,28,29,30,31,32,33,34,35,36,37,38,39^ included in the analysis, the mean (SD) age at baseline was 72.5 (7.5) years and 20 160 participants (58.4%) were female (Table 1). Participants had a mean (SD) 8.2 (5.4) years of education, and the mean (SD) follow-up time was 4.3 (4.3) years. At baseline, the mean (SD) SBP was 138.7 (21.5) mm Hg and DBP was 80.2 (11.3) mm Hg. Of the hypertensive and antihypertensive groups, 10 402 participants (35.5%) were healthy controls, 1293 participants (4.4%) had uncertain hypertension and were excluded, 14 759 participants (50.3%) had treated hypertension, and 2881 participants (9.8%) had untreated hypertension. At baseline, there were 2884 participants with dementia who were excluded from analysis. The mean (SD) time to dementia diagnosis was 4.X (3.5) years, although this metric varied considerably by study (Table 1).

### History of Hypertension and Antihypertensive Use

**Figure 1.**
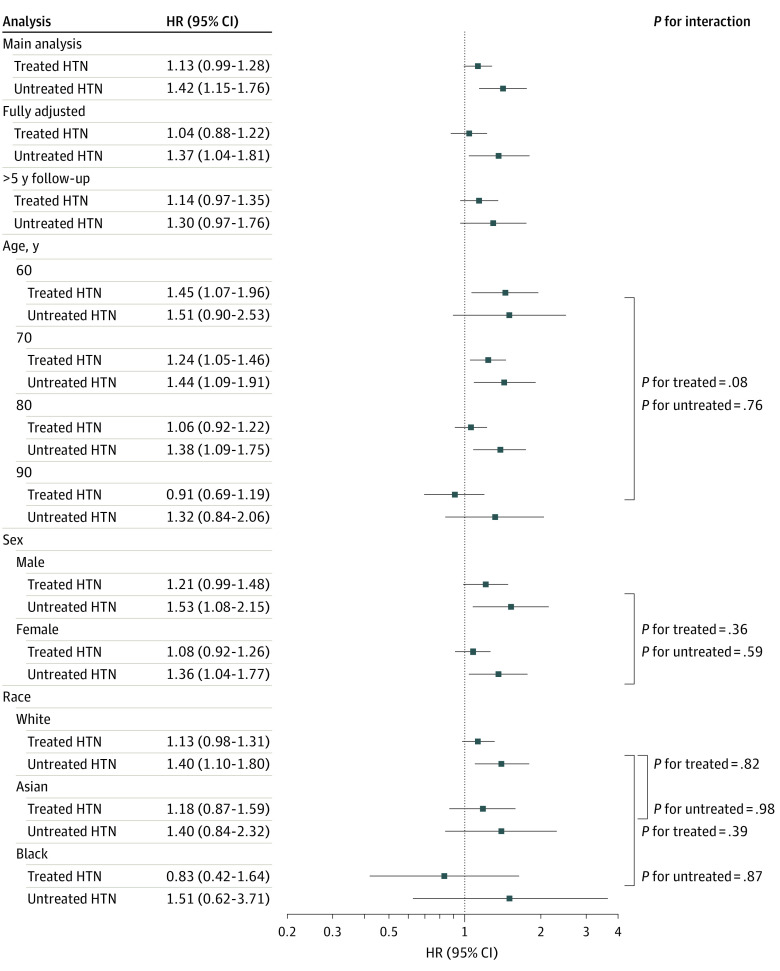
Association of Hypertension (HTN) and Antihypertensive Status With the Risk of All-Cause Dementia The x-axis is in log2 scale. The main analysis (partially adjusted) included covariates of age, age squared, sex, education, and racial group. The fully adjusted analysis included additional covariates of body mass index, smoking status, history of hypercholesterolemia, and diabetes. Each of the other analyses applied the partially adjusted model. The *P* values show the size of the interaction effect for age, sex, and racial group with treated HTN (compared with healthy controls) and untreated HTN (compared with healthy controls). Age was treated as a continuous variable, sex as a categorical variable, and racial group as a categorical variable with 3 major groups (Asian, Black, and White). The *P* values show the significance of the interaction term. The interaction *P* values used White participants as the main comparison group in the racial analysis (as this was the largest group included). HR indicates hazard ratio.

The main analysis in 14 studies^23,24,25,26,29,30,31,32,34,35,36,37,38,39^ found that participants with untreated hypertension had a significantly higher risk of dementia compared with healthy controls (HR, 1.42; 95% CI, 1.15-1.76; *P* = .001) and those with treated hypertension (HR, 1.26; 95% CI, 1.03-1.54; *P* = .03) (Figure 1 and Table 2). There was no significant difference in risk in participants with treated hypertension compared with healthy controls (HR, 1.13; 95% CI, 0.99-1.28; *P* = .07). In the fully adjusted analysis including 9 studies,^23,24,25,26,31,34,35,36,37^ these results were replicated with similar effect sizes, but in the analysis restricted to participants with more than 5 years of follow-up (10 studies^23,25,29,30,31,32,34,35,36,39^), the findings were no longer significant. In the 2-step random-effects meta-analysis, comparisons of treated and untreated hypertension groups showed low heterogeneity of estimates between studies (*I*^2^ = 7.7%), whereas analyses comparing participants with treated and untreated hypertension with healthy controls had a greater level of heterogeneity (treated hypertension: *I*^2^ = 85.6%; untreated hypertension: 57.1%) (eTable 10 in Supplement 1).

**Table 2.** Summary of Cox Proportional Hazards Models Examining Associations of Hypertension and Antihypertensive Status and Baseline Blood Pressure With All-Cause Dementia

Group	Dementia risk					
	Main analysis^a^		Fully adjusted analysis^b^		Restricting to participants with >5 y follow-up	
	HR (95%CI)	*P* value	HR (95%CI)	*P* value	HR (95%CI)	*P* value
Hypertension status^c^						
Treated hypertension (vs healthy controls)	1.13 (0.99-1.28)	.07	1.04 (0.88-1.22)	.64	1.14 (0.97-1.35)	.12
Untreated hypertension (vs healthy controls)	1.42 (1.15-1.76)	.001	1.37 (1.04-1.81)	.03	1.30 (0.97-1.76)	.08
Untreated hypertension (vs treated hypertension)	1.26 (1.03-1.54)	.03	1.32 (1.01-1.72)	.04	1.14 (0.85-1.52)	.37
Baseline blood pressure, mm Hg^d^						
Systolic						
100	1.06 (0.87-1.30)	.94	1.07 (0.82-1.38)	.89	0.91 (0.70-1.20)	.77
120	1.01 (0.94-1.09)		1.00 (0.91-1.10)		0.96 (0.88-1.05)	
140	0.99 (0.95-1.03)		1.01 (0.95-1.06)		1.03 (0.99-1.07)	
160	0.98 (0.90-1.08)		1.00 (0.86-1.15)		1.05 (0.94-1.17)	
180	0.98 (0.88-1.10)		0.93 (0.79-1.10)		1.00 (0.87-1.16)	
Diastolic						
60	1.05 (0.88-1.25)	.16	1.02 (0.78-1.34)	.72	1.06 (0.83-1.34)	.43
70	1.04 (0.97-1.10)		1.01 (0.94-1.08)		1.03 (0.94-1.13)	
80	0.97 (0.94-0.99)		0.98 (0.94-1.01)		0.97 (0.94-1.00)	
90	0.96 (0.90-1.02)		1.00 (0.93-1.08)		0.97 (0.89-1.07)	
100	1.08 (0.97-1.20)		1.11 (0.97-1.28)		1.10 (0.98-1.24)	

Abbreviation: HR, hazard ratio.

^a^ The models were all adjusted for age, age squared, sex, education, and racial group.

^b^ The fully adjusted analysis included additional covariates of body mass index, smoking status, history of hypercholesterolemia, and diabetes.

^c^ The main analysis included 14 studies,^23,24,25,26,29,30,31,32,34,35,36,37,38,39^ 20 381 participants, and 1212 events. The fully adjusted analysis included 9 studies,^23,24,25,26,31,34,35,36,37^ 12 449 participants, and 784 events. Analysis restricted to participants with more than 5 years of follow-up included 10 studies,^23,25,29,30,31,32,34,35,36,39^ 7266 participants, and 669 events.

^d^ The main analysis included 17 studies,^23,24,25,26,27,28,29,30,31,32,33,34,35,36,37,38,39^ 27 508 participants, and 1668 events. The fully-adjusted analysis included 9 studies,^25,26,30,31,34,35,36,38^ 10 589 participants, and 725 events. The analysis restricted to participants with more than 5 years of follow-up included 13 studies,^23,25,27,29,30,31,32,33,34,35,38,39^ 9892 participants, and 887 events. *P* values for the baseline blood pressure natural splines were computed by comparing the fit of the model with and without the natural splines terms.

Interaction analyses found no significant moderation by age, sex, or race (Figure 1; eTable 11 in Supplement 1). Despite there being no significant moderation by age, treated hypertension was associated with increased risk of dementia at ages 60 and 70 years but not at ages 80 or 90 years (Figure 1; eTable 12 in Supplement 1). The greater risk associated with untreated hypertension compared with healthy controls was consistent throughout the various age, sex, and racial groups.

### Baseline BP

**Figure 2.**
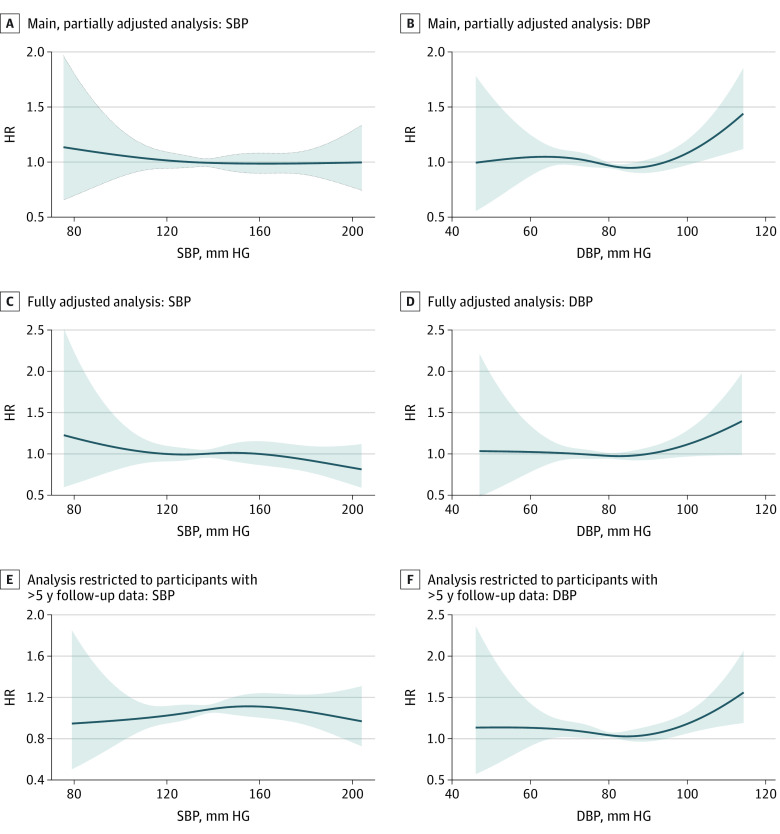
The Associations of Systolic Blood Pressure (SBP) and Diastolic Blood Pressure (DBP) With Dementia Risk In all models, SBP and DBP were centered at the overall mean (SBP: 140 mm Hg; DBP: 80 mm Hg), and all hazard ratios (HRs) represent within-group risk relative to this overall mean; shading indicates 95% CI. A restricted cubic splines model was applied. The partially adjusted analysis included the covariates of age, age squared, sex, education, racial group, and a random effect for study. The fully adjusted analysis included additional covariates of body mass index, smoking status, history of hypercholesterolemia, and diabetes.

In the main, partially adjusted analysis, there were no significant linear or nonlinear associations of baseline SBP or DBP with dementia risk (Table 2 and Figure 2A and B). This finding was confirmed by the fully adjusted model as well as the analysis examining only participants with greater than 5 years of follow-up data (Figure 2C-F). In the 2-step, random-effects meta-analysis, the heterogeneity of the estimates across studies was moderate to small (*I*^2^ for SBP = 24.1%; *I*^2^ for DBP = 46.9%) (eTable 9 in Supplement 1). There were no significant interactions between age, sex, or racial group for SBP or DBP natural splines terms (eTable 13 in Supplement 1). There were no significant interactions between either SBP or DBP and the HT/AHT status of participants indicating their independence (eTable 14 and eTable 15 in Supplement 1).

## Discussion

### Antihypertensive Use and Dementia Risk in Late Life

This IPD meta-analysis found that older adults with untreated hypertension had significantly increased risk of dementia compared with healthy controls and individuals with treated hypertension. Clinical trials examining antihypertensive use in populations with hypertension have tended to find a small association of treatment with reduced risk of dementia.^49^ In the 2022 IPD meta-analysis by Perters et al^20^ of 7 clinical trials with 28 008 participants, participants with treated hypertension had a 13% reduced risk of dementia compared with those with untreated hypertension. Peters et al^20^ stratified for age and found that the largest association was in adults aged 60 to 70 years with hypertension, with no significant associations with antihypertensive use found in adults aged 71 to 80 years and older than 80 years. By contrast, our study found that even at the ages of 70 and 80 years, there was a significantly higher dementia risk in individuals with untreated hypertension compared with the treated hypertension group. A meta-analysis of longitudinal cohort studies by Ou et al,^50^ found that individuals who used antihypertensive in late life (age >65 years) had a 21% lower dementia risk compared with individuals with untreated hypertension, similar to the findings in our study. It is interesting that cohort studies tend to reflect a larger risk difference between treated and untreated hypertension than clinical trials. This may be related to discrepancies in treatment duration, given that patients in cohort studies have been using medications historically (ie, up to many decades), whereas patients in clinical trials have generally been using the medication only for the duration of the trial (eg, only a few years). Alternatively, risk differences between people with treated vs untreated hypertension may be inflated in cohort studies by a number of nonrandom confounders, including poorer health literacy, lower socioeconomic status, greater comorbidity, and reduced access to care.^51^

For participants with treated hypertension, our study found that throughout late life, there was no increased risk of dementia compared with healthy controls, and this result was not significantly altered by age, sex, or race. These results corroborated the findings of a meta-analysis including 71 994 participants by Ou et al^50^ that found no association between late-life hypertension and dementia, a finding also supported by earlier meta-analyses.^52,53^ Whereas some studies have suggested that hypertension was associated with greater dementia risk in Black populations^50^ and in males, our study found no significant differences between racial or sex groups. Epidemiological studies have consistently found greater prevalence of hypertension and vascular disease in Black populations.^54,55^ However, multivariate analyses of cardiovascular outcomes indicate that clinical, environmental, and socioeconomic factors, rather than genetic or intrinsic racial differences, explain cardiovascular differences.^56^ Similarly, our study found that while hypertension exposure was greater in Black populations, its association with dementia risk was not significantly different. This finding is reassuring, insofar as it indicates that the similar treatments are likely to be similarly effective in different racial groups.

### Baseline BP and History of Hypertension and Antihypertensive Use

This study found that baseline SBP and DBP did not significantly modify the association of hypertension and antihypertensive use status with dementia risk. Similarly, a meta-analysis of clinical trials by Peters et al^20^ found that treatment was not modified by baseline systolic BP tertiles, quartiles, or quintiles. By contrast, an IPD meta-analysis of 31 090 adults older than 55 years by Ding et al,^57^ found that antihypertensives were associated with dementia risk reduction only in individuals with high baseline BP and not in the group with BP within reference ranges. The challenge with this bivariate grouping is that it eschews nuance, potentially missing differential associations within smaller BP groups and not capturing nonlinear differences in baseline BP interactions, which our study is able to do. Our findings indicate that baseline BP, being a cross-sectional snapshot of a highly variable^58^ biomarker, is of limited significance when making decisions to continue antihypertensive treatment for dementia risk reduction.

### Late-Life Baseline BP and Dementia Risk

Our study found no significant association between baseline SBP or DBP in late life and dementia risk in any of the analyses. While this corroborates a panoply of previous studies,^50,52,59,60,61,62^ the field remains highly contested, with studies finding that high BP was associated with either an increased^63,64^ or decreased^5,8,18,19^ dementia risk. A number of studies have also found U-shaped associations between dementia risk and BP, but the lowest risk points for these associations vary enormously. In an IPD meta-analysis by Van Dalen et al,^5^ the lowest dementia risk was found at an SBP of 185 mm Hg and a DBP of 139 mm Hg, whereas for the Chicago Health and Aging Project,^6^ the lowest risk was found at an SBP of 138 mm Hg and a DBP of 77 mm Hg.

What sense can be made of these conflicting results, particularly in light of our study? BP in late life is a highly variable biomarker^65,66^ when measured multiple times within a day^67^ or across annual assessments.^68^ Reasons for this include the white coat effect,^69^ interclinician BP measurement differences, diurnal variation, and late-life biological reasons, including poorer autoregulation^70^ and atherosclerosis.^71^ It is also the case that aging-associated vascular calcification and atherosclerosis make BP, and particularly SBP, less reflective of central BP,^72^ which is the measure of greatest significance to cerebral health. The second consideration is that high or low late-life BP will affect individuals differently depending on their vascular history, such as whether midlife hypertension is followed by late life hypotension or hypertension.^73,74,75^ A third consideration is reverse causality. Some argue that lower BPs in late life may cause dementia and cognitive decline, but there is good evidence that dementia and the associated loss of central vascular control, weight loss, and ill health may cause lower BPs.^70^ Given these complex and not fully understood effects, it is unsurprising that there has been such a diversity of results. An IPD meta-analysis by Peters et al^20^ found that BP reduction in late life was associated with diminished dementia risk down to an SBP of 100 mm Hg and a DBP of 70 mm Hg. Our study, in combination with these results, provides the strongest data yet for the importance of antihypertensive use even in late life^21,22^ and that more than a single late-life BP measure is needed to guide risk stratification and treatment decisions.

### Limitations

This study has some limitations, with the primary limitation being variability in cohort study design. Definitions of hypertension change over time and vary across locations, leading to potential differences in diagnosis. Similarly, the cohort studies we included varied in the cognitive instruments and criteria for dementia used (including *DSM-III-R*, *DSM-IV* and *International Statistical Classification of Diseases and Related Health Problems, Tenth Revision* [*ICD-10*]). Furthermore, studies with more regular follow up periods likely capture a more accurate date of dementia onset, which may have also altered results by modifying the length of diagnosis in various studies. The brief time to onset of dementia in some of the studies suggests the presence of baseline cognitive impairment. Individuals in the early stages of dementia may engage less with medical services or forget to take medications; thus, the cognitive impairment may contribute to undertreatment of hypertension rather than the reverse. We did not have the data to control for some confounders that may contribute to differences in dementia risk between treated and untreated hypertension, including socioeconomic status and poor management of other health conditions. Clearly, individuals with better health literacy and more access to medications will have a panoply of other differences that may contribute to reduced dementia risk. We also did not have data on competing events to dementia, such as death and stroke, which may also modify its association with antihypertensive use. Our study did not provide detail on the classes or doses of antihypertensives used. Previous studies have indicated that angiotensin II receptor blockers may reduce dementia risk more than other types of antihypertensives,^76^ but we did not have information on antihypertensive class to investigate this potentially important moderating factor. Additionally, our classification of participants into 4 broad racial groups does not consider the ethnic and genetic diversity that exists within each of these.

## Conclusion

In this IPD meta-analysis with data from 16 countries, we found that hypertension was a risk factor associated with dementia in late life. Antihypertensive use was associated with decreased dementia risk in late-life individuals with hypertension; thus, dementia risk reduction may be 1 of the multiple goals of antihypertensive treatment in late-life (eg, prevention of ischemic heart disease, chronic kidney disease). A single measure of SBP or DBP at baseline had no significant association with late-life dementia risk, and, corroborating extant hypertension guidelines,^46^ it seems that more than 1 measure is needed to inform treatment.

## Notes

Supplement 1.
eMethods.
**eTable 1.** Ethics Approvals for Studies **eTable 2.** Missingness and Dementia **eTable 3.** Covariates in Studies **eTable 4.** Comparing Covariates of the Hypertension Groups **eTable 5.** Methods of Blood Pressure Measurement **eTable 6.** Year Values Assigned to Educational Attainment, Harmonisation of Education Variables **eTable 7.** Diabetes and Hypercholesterolemia Study Specific Details and Harmonization **eTable 8.** Diagnostic Criteria and Method of Diagnosis for Dementia in Each Study **eTable 9.** Hypertension History in Treated Compared With Untreated Populations **eTable 10.** Two-Step Meta-Analysis **eTable 11.** Interactions Between HT/AHT Status and Age, Sex, and Ethnicity Groups **eTable 12.** Robustness of the HT/AHT Status Results in Later Age Groups **eTable 13.** Interactions Between SBP/DBP and Age, Sex, and Ethnicity Groups **eTable 14.** Interaction Terms Between HT/AHT Status and BP **eTable 15.** HT/AHT Status and Dementia, Including Those With BP >160/100 mm Hg in the Untreated Hypertension Group

Supplement 2.
Data Sharing Statement
