# Supplementary material for: Use of Antihypertensives, Blood Pressure, and Estimated Risk of Dementia in Late Life: An Individual Participant Data Meta-Analysis
Source: JAMA Netw Open. 2023 Sep 12;6(9):e2333353. doi: 10.1001/jamanetworkopen.2023.33353 (PMC10498335; doi:10.1001/jamanetworkopen.2023.33353)
Supplement: Supplement 1. — eMethods. eTable 1. Ethics Approvals for Studies eTable 2. Missingness and Dementia eTable 3. Covariates in Studies eTable 4. Comparing Covariates of the Hypertension Groups eTable 5. Methods of Blood Pressure Measurement eTable 6. Year Values Assigned to Educational Attainment, Harmonisation of Education Variables eTable 7. Diabetes and Hypercholesterolemia Study Specific Details and Harmonization eTable 8. Diagnostic Criteria and Method of Diagnosis for Dementia in Each Study eTable 9. Hypertension History in Treated Compared With Untreated Populations eTable 10. Two-Step Meta-Analysis eTable 11. Interactions Between HT/AHT Status and Age, Sex, and Ethnicity Groups eTable 12. Robustness of the HT/AHT Status Results in Later Age Groups eTable 13. Interactions Between SBP/DBP and Age, Sex, and Ethnicity Groups eTable 14. Interaction Terms Between HT/AHT Status and BP eTable 15. HT/AHT Status and Dementia, Including Those With BP >160/100 mm Hg in the Untreated Hypertension Group [file jamanetwopen-e2333353-s001.pdf]

Supplemental Online Content

Lennon MJ, Lam BP, Lipnicki DM, et al. Use of antihypertensives, blood pressure, and estimated risk of dementia in late life: an individual participant data meta-analysis. *JAMA Netw Open*. 2023;6(9):e2333353. doi:10.1001/jamanetworkopen.2023.33353

eMethods.
eTable 1. Ethics Approvals for Studies
eTable 2. Missingness and Dementia
eTable 3. Covariates in Studies
eTable 4. Comparing Covariates of the Hypertension Groups
eTable 5. Methods of Blood Pressure Measurement
eTable 6. Year Values Assigned to Educational Attainment, Harmonisation of Education Variables
eTable 7. Diabetes and Hypercholesterolaeamia Study Specific Details and Harmonization
eTable 8. Diagnostic Criteria and Method of Diagnosis for Dementia in Each Study
eTable 9. Hypertension History in Treated Compared With Untreated Populations
eTable 10. Two-Step Meta-Analysis
eTable 11. Interactions Between HT/AHT Status and Age, Sex, and Ethnicity Groups
eTable 12. Robustness of the HT/AHT Status Results in Later Age Groups
eTable 13. Interactions Between SBP/DBP and Age, Sex, and Ethnicity Groups
eTable 14. Interaction Terms Between HT/AHT Status and BP
eTable 15. HT/AHT Status and Dementia, Including Those With BP >160/100 mm Hg in the Untreated Hypertension Group

This supplemental material has been provided by the authors to give readers additional information about their work.

© 2023 Lennon MJ et al. *JAMA Network Open.*

eMethods

Categorisation of covariates

Education level was provided either as years of education or in a categorical form that was converted to number of years (Table S4) and treated as a continuous variable. Racial group was treated as a 4-level categorical variable (0–White, 1–Asian, 2–Black, 3–Other). Other covariates included body mass index (BMI) (continuous variable), diabetes status (categorical variable; 0–no diabetes, 1–diabetes), hypercholesterolaemia (categorical variable; 0–no hypercholesterolaemia, 1–hypercholesterolaemia) and smoking status (categorical variable; 0–never smoker, 1–previous smoker, 2–current smoker).

Removal of the "Uncertain Hypertension" group

Those not reporting hypertension history but taking an antihypertensive were considered anomalous for several reasons: 1. They may have been taking an antihypertensive but not been aware or failed to recall that they had been diagnosed with hypertension previously 2. They may have been taking an antihypertensive for a reason other than hypertension (e.g. heart failure, palpitations, arrhythmias, kidney disease). It is for these reasons that they were removed from the relevant analyses.

Sensitivity analyses for those with elevated BP

In the paper we tested the interaction between HT/AHT status and baseline blood pressure. However, given that those who have markedly elevated BP at baseline are likely to have true hypertension rather than it being a timing or setting effect, we included a supplementary analysis that categorised those reporting no history of hypertension but with an SBP ≥ 160 mmHg or DBP ≥ 100 into the "Untreated hypertension" group. In this sensitivity analysis we found that the untreated hypertension group had significantly higher dementia risk compared to the "healthy controls" (HR=1.26, 95%CI[1.04, 1.53], p=0.019) but not the "treated hypertension" group (HR=1.14, 95%CI[0.96, 1.36], p=0.14) (Table S16).

Assessing the robustness of the age analysis

Age was included in the interaction term as a continuous variable and estimates of the effects at different age levels (60, 70, 80 and 90) were computed by centring age at each of these levels and re-running the analysis. However, to ensure that non-linear effects of age were not missed restricted analyses were run in those older than 65, 75 and 85 years. In this sensitivity analysis for older age groups, the "untreated hypertension" group had significantly higher dementia risk compared to those with "treated hypertension" when restricting to those ≥65 (HR=1.29, 95%CI[1.02, 1.62], p=0.031), ≥75 (HR=1.39, 95%CI[1.04, 1.85], p=0.026) and ≥85 years old (HR=1.85, 95%CI[1.06, 3.22], p=0.031) (Table S13).

© 2023 Lennon MJ et al. *JAMA Network Open.*

eTable 1. Ethics Approvals for Studies

Study	Institutional Review Board
**CLAS**	The National Pillar Program of China Ministry of Science and Technology (project number:2009BAI77B03) and The National Key Clinical Disciplines at Shanghai Mental Health Center (Office of Medical Affairs, China Ministry of Health, 2011-873; OMA-MH, 2011-873).
**EAS**	Albert Einstein College of Medicine Institutional Review Board (Approval#1996-175)
**EPIDEMCA**	Approved by Congolese ethical committee CERSSA (Comité d'Ethique de la Recherche en Sciences de Santé) and by an ethics review board (Comité de Protection des Personnes Sud-Ouest Outre Mer) in France.
**ESPRIT**	Ethics committee (CCPPRB) of the Kremlin Bicetre hospital (n° registered 99-28)
**H70**	The H70 study was conducted according to the ethical standards of the institutional committee and with the 1964 Helsinki Declaration and its later amendments or comparable ethical standards. Approved by the Regional Ethical Review Board in Gothenburg (approval numbers: S227-00, T453-04, 075-09, 131-15, and 278-18).
**HELIAD**	All procedures were approved by the Insti- tutional Ethics Review Board of the University of Thessaly.
**ISA**	The survey was approved by the University of Ibadan and University College Hospital, Ibadan joint ethical review board.
**Indi_ibadan**	Institutional Ethics Review Board of Indiana University, Indianapolis and University of Ibadan, Ibadan
**Invece.Ab**	Ethics Committee of the University of Pavia (#3/2009)
**KLOSCAD**	Institutional Review Board of Seoul National University Bundang Hospital, Korea (IRB No. B-0912/089-010)
**LEILA75+**	Ethics committee of the University of Leipzig (C7 79934700)
**PATH**	Australian National University Human Research Ethics Committee (#M9807, #2002/189, #2006/314, # 2010/542, #2001/2, #2009/039)
**SALSA**	University of California, San Francisco Human Research Protection Program Institutional Review Board (IRB#10-00243)
**SLASI**	National University of Singapore Institutional Review Board (Reference Code: 04-140)
**SPAH**	Ethical Committee for the Analysis of Research Projects (CAPesq) - Hospital das Clínicas and Medical School - Project Registry Number: 257/2002; National Ethical Committee on Research (CONEP-Brazil) - Project Registry Number: 4355
**Sydney MAS**	University of New South Wales Human Research Ethics Committee (approval #14327)
**ZARADEMP**	Ethics committee of the Zaragoza University Hospital (CEICA # CP16/2012)

Ethics approvals for the studies included in the analysis.

© 2023 Lennon MJ et al. *JAMA Network Open.*

eTable 2. Missingness and Dementia

Study	Total N	Baseline Dementia N (%)	SBP outliers N (%)	DBP outliers N (%)	Model 1 N (%)	Model 2 N (%)
**CLAS**	3246	350 (10.8%)	2 (0.1%)	3 (0.1%)	2111 (65%)	0 (0%)
**EPIDEMCA**	2002	270 (13.5%)	68 (3.4%)	49 (2.4%)	321 (16%)	178 (8.9%)
**HELIAD**	1999	99 (5%)	2 (0.1%)	1 (0.1%)	1877 (93.9%)	0 (0%)
**Ibadan**	4261	64 (1.5%)	120 (2.8%)	78 (1.8%)	1650 (38.7%)	0 (0%)
**Indianapolis**	3992	105 (2.6%)	12 (0.3%)	3 (0.1%)	1455 (36.4%)	0 (0%)
**EAS**	2255	132 (5.9%)	0 (0%)	0 (0%)	2055 (91.1%)	341 (15.1%)
**ESPRIT**	2259	69 (3.1%)	6 (0.3%)	4 (0.2%)	2188 (96.9%)	2126 (94.1%)
**GothenburgH70**	1221	102 (8.4%)	18 (1.5%)	8 (0.7%)	786 (64.4%)	0 (0%)
**Invece.Ab**	1321	39 (3%)	2 (0.2%)	1 (0.1%)	1269 (96.1%)	1200 (90.8%)
**KLOSCAD**	6833	349 (5.1%)	0 (0%)	11 (0.2%)	6169 (90.3%)	3263 (47.8%)
**LEILA**	1265	220 (17.4%)	41 (3.2%)	45 (3.6%)	985 (77.9%)	0 (0%)
**PATH**	2551	8 (0.3%)	5 (0.2%)	9 (0.4%)	2536 (99.4%)	2167 (84.9%)
**SALSA**	1789	68 (3.8%)	8 (0.4%)	11 (0.6%)	1696 (94.8%)	1484 (83%)
**SLASI**	2804	113 (4%)	5 (0.2%)	4 (0.1%)	391 (13.9%)	390 (13.9%)
**SPAH**	2072	105 (5.1%)	45 (2.2%)	66 (3.2%)	1818 (87.7%)	0 (0%)
**SydneyMAS**	1037	0 (0%)	7 (0.7%)	4 (0.4%)	1037 (100%)	945 (91.1%)
**ZARADEMP**	4803	223 (4.6%)	16 (0.3%)	25 (0.5%)	4376 (91.1%)	0 (0%)
**ISA**	2149	340 (15.8%)	71 (3.3%)	30 (1.4%)	1799 (83.7%)	0 (0%)
**Total**	56821	2884 (5.1%)	481 (0.8%)	391 (0.7%)	34519 (60.8%)	12094 (21.3%)

Total numbers of participants in each of the studies. Dementia column shows the number and percent of participants with dementia at baseline who were excluded from the study. The SBP and DBP columns show numbers and percentages of individuals who were removed from the baseline BP analysis for having a BP outside three standard deviations from the mean. The final two columns to the right show the numbers of percentages of participants who had data that was sufficiently complete to be included in the partially (model 1) and fully (model 2) adjusted analysis.

© 2023 Lennon MJ et al. *JAMA Network Open.*

eTable 3. Covariates in Studies

Study	Mean BMI (SD)	Diabetes (%)	High Cholesterol (%)	Smoking Status (N, %)
**CLAS**	23.7 (3.5)	17.30%	24.80%	1 - 1442 (68.3%) 2 - 353 (16.7%) 3 - 316 (15%)
**EPIDEMCA**	20.9 (4.3)	7%	8.10%	1 - 238 (74.1%) 2 - 17 (5.3%) 3 - 66 (20.6%)
**HELIAD**	-	17.40%	41.70%	1 - 1140 (61%) 2 - 524 (28%) 3 - 206 (11%)
**Ibadan**	21.4 (4.5)	2.10%	-	1 - 1138 (69%) 2 - 512 (31%) 3 - 0 (0%)
**Indianapolis**	29.5 (5.8)	26.40%	-	1 - 593 (40.8%) 2 - 859 (59.2%) 3 - 0 (0%)
**EAS**	28.3 (5.4)	18%	15.50%	1 - 919 (45%) 2 - 977 (47.9%) 3 - 144 (7.1%)
**ESPRIT**	25.1 (3.7)	9.30%	56.50%	1 - 1266 (57.9%) 2 - 775 (35.4%) 3 - 146 (6.7%)
**GothenburgH70**	-	9.10%	67%	1 - 317 (47.7%) 2 - 257 (38.7%) 3 - 90 (13.6%)
**Invece.Ab**	27.2 (4.6)	17.60%	33.20%	1 - 730 (57.5%) 2 - 418 (32.9%) 3 - 121 (9.5%)
**KLOSCAD**	24 (3)	28.40%	47.30%	1 - 4274 (69.6%) 2 - 1157 (18.8%) 3 - 714 (11.6%)
**LEILA**	-	22.80%	-	1 - 659 (67.2%) 2 - 256 (26.1%) 3 - 65 (6.6%)
**PATH**	26.9 (5.3)	7.50%	22.80%	1 - 1319 (52%) 2 - 942 (37.2%) 3 - 274 (10.8%)
**SALSA**	29.8 (5.7)	32.40%	51.60%	1 - 777 (45.9%) 2 - 725 (42.8%) 3 - 192 (11.3%)
**SLASI**	23.9 (3.4)	13.80%	51.40%	1 - 327 (83.6%) 2 - 36 (9.2%) 3 - 28 (7.2%)
**SPAH**	25.8 (4.8)	22.60%	31.90%	1 - 759 (76%) 2 - 0 (0%) 3 - 240 (24%)
**SydneyMAS**	27.1 (4.5)	15.60%	68.30%	1 - 476 (46%) 2 - 526 (50.8%) 3 - 33 (3.2%)
**ZARADEMP**	26.9 (5)	12.70%	-	1 - 2867 (65.6%) 2 - 938 (21.5%) 3 - 565 (12.9%)
**ISA**	-	2.10%	-	
**Total**	25.8 (4.8)	16.90%	41.40%	1 - 19241 (60.7%) 2 - 9272 (29.2%) 3 - 3200 (10.1%)

Summary of the prevalence of co-variate risk factors in each of the studies. Diabetes and high cholesterol harmonisation details can be seen in Table S5. For smoking status 1 – “Never smoker”, 2 – “Past smoking” and 3 – “Current smoking”.

© 2023 Lennon MJ et al. *JAMA Network Open.*

eTable 4. Comparing Covariates of the Hypertension Groups

	"Healthy Controls"	Uncertain Hypertension	Treated Hypertension	Untreated Hypertension	P values
**Mean Age (SD)**	70.1 (7)	75.6 (7.1)	72.6 (7.4)	71.5 (7.4)	**<0.001**
**Sex (M%)**	43.80%	47.30%	41%	43.50%	**<0.001**
**Mean Education Yrs (SD)**	9.6 (5.2)	9.8 (4.9)	8.4 (5.2)	6.6 (5.5)	**<0.001**
**Mean FU Yrs (SD)**	5.5 (5)	5.3 (4.9)	4.3 (4.2)	4.7 (4.8)	**<0.001**
**No. Dementia (%)[a]**	456 (4.4%)	93 (7.2%)	660 (4.5%)	123 (4.3%)	0.69
**Mean time to Dementia (SD)**	5 (3.8)	4.3 (3.4)	3.7 (3.2)	2.8 (2.6)	**<0.001**
**Mean SBP (SD) (mmHg)**	130.9 (17.7)	130.5 (17.1)	140.3 (20)	148.7 (21.5)	**<0.001**
**Mean DBP (SD) (mmHg)**	78.1 (9.6)	75.2 (9.5)	80.7 (11.1)	85.1 (12.1)	**<0.001**
**BMI**	24.7 (4.3)	25.8 (4.4)	26.6 (4.9)	25.7 (5.2)	**<0.001**
**Diabetes (%)**	10.60%	19.70%	24.40%	17.20%	**<0.001**
**High Cholesterol (%)**	35.40%	47.80%	50.70%	38.10%	**<0.001**
**Smoking Status (N, %)**	1 - 5857 (59.8%)<br>2 - 2706 (27.6%)<br>3 - 1230 (12.6%)	1 - 581 (48.9%)<br>2 - 494 (41.5%)<br>3 - 114 (9.6%)	1 - 8414 (61.8%)<br>2 - 3981 (29.3%)<br>3 - 1211 (8.9%)	1 - 1495 (59.5%)<br>2 - 597 (23.7%)<br>3 - 422 (16.8%)	**<0.001**

Comparison of the four hypertension history/antihypertensive use groups. Because the uncertain hypertension group were excluded from the analysis the p-values on the side represent an assessment of the significance of the difference between "Healthy Controls", Treated Hypertension and Untreated Hypertension. The groups were concatenated from the 14 studies with available data. These included: CLAS, EAS, EPIDEMCA, ESPRIT, H70, HELIAD, Invece.Ab, KLOSCAD, PATH, SALSA, SLASI, SPAH, SydneyMAS and ZARADEMP. For continuous variables comparison was made by 2-tailed ANOVA and for categorical variables chi-squared analysis was used.

© 2023 Lennon MJ et al. *JAMA Network Open.*

eTable 5. Methods of Blood Pressure Measurement

Abbreviation	Blood Pressure Method Description	Position	Number of Measures taken at baseline
**CLAS**	-	Sitting	1
**EPIDEMCA**	-	Sitting	4
**EAS**	Blood pressure is measured using a standard mercury sphygmomanometer, appropriate sized cuff and stethoscope. It is done on the right arm while participant is sitting.	Sitting	2
**ESPRIT**	Automatic Blood pressure taken using digital electronic tensiometer OMRON M4	Sitting	2
**H70**	Systolic and diastolic blood pressure were recorded in the right arm in the sitting position after 5 min rest using a standard cuff (Umedico) - a manual sphygmomanometer (manual blood pressure cuff).	Sitting	1
**HELIAD**	Measured once manually by the examining neurologist	Sitting	1
**ISA**	Participants had to be seated for at least 5 min before the measurements. This was done using an electronic sphygmomanometer. Both the diastolic and systolic BP were recorded twice on the left arm with five minutes interval between the two measurements. The average of the two measures was used.	Sitting	2
**indi_ibadan**	Two consecutive measurements of systolic and diastolic blood pressure were taken by trained interviewers using a mercury sphygmomanometer while the participant was seated	Sitting	2
**Invece.Ab**	Blood pressure is measured using a standard mercury sphygmomanometer, an appropriately sized cuff, and a stethoscope. It is performed during the medical examination after taking the medical history, on the left arm while the participant is lying down for 5 minutes on the medical examination table	Lying down with head at angle	3
**KLOSCAD**	A standard mercury sphygmomanometer, appropriate sized cuff and stethoscope (every centers at baseline) or Automatic Blood pressure (recently, some centers)	Sitting	3
**LEILA75+**	Automatic Blood pressure	Sitting	1

© 2023 Lennon MJ et al. *JAMA Network Open.*

**PATH**	Automatic Blood pressure	Sitting	2
**SAS**	-	Sitting	2
**SALSA**	-	Sitting	1
**SLASI**	-	Sitting	1
**SPAH**	-	Sitting	3
**Sydney MAS**	Automatic Blood pressure	Sitting	3
**ZARADEMP**	A standard mercury sphygmomanometer (Manual), appropriate sized cuff and stethoscope	Sitting	1

Information on the method of blood pressure measurement in each of the studies

© 2023 Lennon MJ et al. *JAMA Network Open.*

eTable 6. Year Values Assigned to Educational Attainment, Harmonisation of Education Variables

Study	Category	Years
EPIDEMCA	Never attended school	0
	Schooled but never completed primary	3
	Primary completed	6
	Secondary completed	12
	Tertiary or higher education	16
ESPRIT	None	0
	Primary	5
	1st to 4th year senior school/higher primary	9
	Short technical or professional	11
	5th year to upper 6th form	12
	Long technical or professional	12
	Higher education including higher technical education	15
Indi_ibadan		
Ibadan	Went to School (binary)	Assigned 10 years (average number of years of school for those who attend school)
Indianapolis	Provides number of years of schooling	

Year values assigned to categorical groupings of educational attainment in various studies.

© 2023 Lennon MJ et al. *JAMA Network Open.*

eTable 7. Diabetes and Hypercholesterolaeamia Study Specific Details and Harmonization

Study	Diabetes	Hypercholesterolaemia
**CLAS**	History	History
**EPIDEMCA**	1. History 2. Treatment 3. High sugar level	1. Cholesterol (High Cholesterol Level (>5.3mmol/L))
**EAS**	History	1. Cholesterol, 2. Triglycerides
**ESPRIT**	1. Treatment, 2. Fasting blood glucose	1. Treatment, 2. Cholesterol, 3. Triglycerides
**H70**	1. Treatment, 2. Fasting blood glucose	1. Treatment, 2. Cholesterol, 3. Triglycerides
**HELIAD**	History	History
**ISA**	History	
**indi_ibadan**	History	
**Invece.Ab**	1. Treatment, 2. History	1. Treatment, 2. History
**KLOSCAD**	1. History (also having follow-up current status data or age first diagnosed/began medication), 2. Self-reported current, 3. Fasting blood glucose, 4. Non-fasting blood glucose ≥200mg/dL	1. History (also having follow-up current status data or age first diagnosed/began medication), 2. Self-reported current, 3. Cholesterol, 4. Triglycerides
**PATH**	1. History, 2. Treatment	1. Treatment
**LEILA75+**	Self-reported	
**SAS**	Self-reported history of diagnosis	Self-reported history of diagnosis
**SALSA**	1. Self-report, 2. Fasting blood glucose, 3. Medication	1. Medication, 2. Cholesterol, 3. Triglycerides

© 2023 Lennon MJ et al. *JAMA Network Open.*

**SGS**	Self-reported history of diagnosis	Self-reported history of diagnosis
**SLASI**	1. Fasting blood glucose, 2. Treatment, 3. History	1. Treatment, 2. History, 3. Triglycerides
**SPAH**	1. Fasting blood glucose, 2. Treatment	1. Cholesterol, 2. Triglycerides
**Sydney MAS**	1. Fasting blood glucose, 2. Treatment, 3. History	1. Treatment, 2. History, 3. Cholesterol, 4. Triglycerides
**Tajiri**	1. Fasting blood glucose, 2. Treatment (diet)	1. Cholesterol, 2. Triglycerides, 3. Treatment
**ZARADEMP**	Diagnosis using EURODEM Risk Factor Questionnaire and medical records	

© 2023 Lennon MJ et al. *JAMA Network Open.*

eTable 8. Diagnostic Criteria and Method of Diagnosis for Dementia in Each Study

Study	Criteria
**CLAS**	DSM-IV
**EAS**	DSM-IV
**EPIDEMCA**	DSM-IV and NINCDS-ADRDA criteria were required for dementia and Alzheimer's disease (AD) diagnoses respectively
**ESPRIT**	Standardized interview by a neurologist incorporating cognitive testing, with diagnoses validated by an independent panel of expert neurologists
**H70**	DSM-III-R
**HELIAD**	DSM-IV-TR/NINDS
**ISA**	DSM-IV and the ICD-10 criteria
**indi_ibadan**	DSM-III-R and ICD-10
**Invece.Ab**	DSM-IV
**KLOSCAD**	DSM-IV
**PATH**	DSM-IV
**LEILA75+**	DSM-IV
**SALSA**	California ADDTC criteria for vascular dementia and NINDS-ADRDA for Alzheimer's disease
**SLASI**	DSM-IV
**SPAH**	DSM-IV
**Sydney MAS**	DSM-IV
**ZARADEMP**	DSM-IV

Diagnostic criteria applied for dementia diagnoses in included studies.

© 2023 Lennon MJ et al. *JAMA Network Open.*

eTable 9. Hypertension History in Treated Compared With Untreated Populations

	Main Analysis (n = 32,061, nevent = 2,136)		Fully Adjusted Analysis (n = 13,849, nevent = 850)		Restricting to >5 years FU (n = 11,392, nevent = 1,146)	
	HR (95% CI)	P	HR (95% CI)	P	HR (95% CI)	P
**Hypertension History (Y v N)**	1.10 (1.00, 1.20)	**0.05**	1.12 (0.97, 1.29)	0.14	1.14 (1.00, 1.29)	**0.04**
	**Main Analysis (n = 20,381, nevent = 1,212)**		**Fully Adjusted Analysis (n = 12,449, nevent = 784)**		**Restricting to >5 years FU (n = 7,266, nevent = 669)**	
**Treated hypertension (comp “healthy controls”)**	1.13 (0.99, 1.28)	0.07	1.04 (0.88, 1.22)	0.64	1.14 (0.97, 1.35)	0.12
**Untreated hypertension (comp “healthy controls”)**	1.42 (1.15, 1.76)	**0.001**	1.37 (1.04, 1.81)	**0.03**	1.30 (0.97, 1.76)	0.08
**Untreated hypertension (comp Treated hypertension)**	1.26 (1.03, 1.54)	**0.02**	1.32 (1.01, 1.72)	**0.04**	1.14 (0.85, 1.52)	0.37

Summary of Cox Proportional Hazards Models examining relationship between Hypertension history, HT/AHT status and dementia. The models were adjusted for Age, Age^2, Sex, Education and Ethnicity. There were 17 studies included in the main analysis (CLAS, EAS, EPIDEMCA, ESPRIT, H70, HELIAD, Indi_ibadan, Invece.Ab, ISA, KLOSCAD, LEILA75+, PATH, SALSA, SLASI, SPAH, SydneyMAS and ZARADEMP). There were 9 studies included in the fully adjusted model (EAS, EPIDEMCA, ESPRIT, Invece.Ab, KLOSCAD, PATH, SALSA, SLASI and SydneyMAS). The fully adjusted analysis included additional covariates of BMI, smoking status, history of hypercholesterolaemia and diabetes. There were 13 studies included in the >5 year follow up analysis (CLAS, EAS, ESPRIT, H70, HELIAD, Ibadan, Indianapolis, KLOSCAD, LEILA75+, PATH, SALSA, SydneyMAS and ZARADEMP). The results show that when patients with hypertension are split into treated and untreated hypertension there are considerably different effect sizes and p-values.

© 2023 Lennon MJ et al. *JAMA Network Open.*

eTable 10. Two-Step Meta-Analysis

	HR (95% CI)	P	I2 (%)	Tau2
**HT/AHT Status (n = 20,381, nevent = 1,212)**				
**Treated Hypertension (comp "Healthy Controls")**	1.01 (1.38, 0.73)	0.97	85.6	0.206
**Untreated Hypertension (comp "Healthy Controls")**	1.11 (1.55, 0.79)	0.55	57.1	0.152
**Untreated Hypertension (comp Treated Hypertension)**	1.10 (1.36, 0.89)	0.38	7	0.006
**Baseline Blood Pressure (n = 27,508, nevent = 1,168)**				
**SBP**	0.98 (1.00, 0.96)	0.14	24.5	0
**SBP^2**	1.00 (1.00, 1.00)	0.60	19.2	0
**DBP**	0.98 (1.02, 0.93)	0.26	46.9	0.002
**DBP^2**	1.00 (1.01, 0.99)	0.74	24.9	0

Summary of 2 Step Random Effects Meta-analysis of Cox Proportional Hazards Models run in each study individually. The key variables were HT/AHT status, SBP, DBP and Dementia. The models were adjusted for Age, Age^2, Sex, Education and Ethnicity. There were 14 studies included in the main analysis for HT/AHT status (CLAS, EAS, EPIDEMCA, ESPRIT, H70, HELIAD, Invece.Ab, KLOSCAD, PATH, SALSA, SLASI, SPAH, SydneyMAS and ZARADEMP). There were 17 studies included in the main analysis for SBP and DBP (CLAS, EAS, EPIDEMCA, ESPRIT, H70, HELIAD, Indi_ibadan, Invece.Ab, ISA, KLOSCAD, LEILA75+, PATH, SALSA, SLASI, SPAH, SydneyMAS and ZARADEMP).

© 2023 Lennon MJ et al. *JAMA Network Open.*

eTable 11. Interactions Between HT/AHT Status and Age, Sex, and Ethnicity Groups

Interactions between HT/AHT status and Age, Sex, Ethnicity (n = 21,737, nevent = 2,173)		
	**HR (95% CI)**	**P**
**Age*Interaction**		
**Age**	1.90 (1.74, 2.08)	**<0.001**
**Treated Hypertension (comp "Healthy Controls")**	1.17 (1.03, 1.34)	**0.02**
**Untreated Hypertension (comp "Healthy Controls")**	1.42 (1.13, 1.79)	**0.003**
**Untreated Hypertension (comp Treated Hypertension)**	1.21 (0.97, 1.51)	0.09
**Treated Hypertension*Age (comp "Healthy Controls")**	0.93 (0.85, 1.01)	0.08
**Untreated Hypertension*Age (comp "Healthy Controls")**	0.98 (0.85, 1.13)	0.76
**Untreated Hypertension*Age (comp Treated Hypertension)**	1.06 (0.92, 1.22)	0.43
**Sex interaction**		
**Sex**	1.02 (0.84, 1.25)	0.81
**Treated Hypertension (comp "Healthy Controls")**	1.08 (0.92, 1.26)	0.36
**Untreated Hypertension (comp "Healthy Controls")**	1.36 (1.04, 1.77)	**0.02**
**Untreated Hypertension (comp Treated Hypertension)**	1.26 (0.98, 1.61)	0.07
**Treated Hypertension*Sex (comp "Healthy Controls")**	1.13 (0.88, 1.45)	0.36
**Untreated Hypertension*Sex (comp "Healthy Controls")**	1.13 (0.74, 1.72)	0.59
**Untreated Hypertension*Sex (comp Treated Hypertension)**	1.00 (0.66, 1.51)	1.00
**Ethnicity Interaction**		
**Ethnicity (Asian)**	0.76 (0.23, 2.47)	0.65
**Ethnicity (Black)**	1.63 (0.89, 3.01)	0.11
**Treated Hypertension (comp "Healthy Controls")**	1.13 (0.98, 1.31)	0.10
**Untreated Hypertension (comp "Healthy Controls")**	1.41 (1.10, 1.80)	**0.006**
**Untreated Hypertension (comp Treated Hypertension)**	1.24 (0.98, 1.57)	0.07
**Treated Hypertension*Ethnicity (Asian) (comp "Healthy Controls")**	1.04 (0.75, 1.45)	0.82
**Treated Hypertension*Ethnicity (Black) (comp "Healthy Controls")**	0.74 (0.37, 1.47)	0.39
**Untreated Hypertension*Ethnicity (Asian) (comp "Healthy Controls")**	0.99 (0.56, 1.75)	0.98
**Untreated Hypertension*Ethnicity (Black) (comp "Healthy Controls")**	1.08 (0.43, 2.73)	0.87
**Untreated Hypertension*Ethnicity (Asian) (comp Treated Hypertension)**	0.96 (0.57, 1.62)	0.86
**Untreated Hypertension*Ethnicity (Black) (comp Treated Hypertension)**	1.46 (0.66, 3.23)	0.36

Summary of Cox Proportional Hazards Models examining the interaction between HT/AHT status and Age, Sex and Ethnicity. The models were adjusted for Age, Age^2, Sex, Education, Racial group and a random intercept for Study. There were 14 studies included in the main analysis for HT/AHT status (CLAS, EAS, EPIDEMCA, ESPRIT,H70, HELIAD, Invece.Ab, KLOSCAD, PATH, SALSA, SLASI, SPAH, SydneyMAS and ZARADEMP).

© 2023 Lennon MJ et al. *JAMA Network Open.*

eTable 12. Robustness of the HT/AHT Status Results in Later Age Groups

	Restricted Analysis to ≥ 65 years old (n = 14,737, nevent = 977)		Restricted Analysis to ≥ 75 yo (n = 5387, nevent = 576)		Restricted Analysis to ≥ 85 yo (n = 854, nevent = 119)	
	**HR (95% CI)**	**P**	**HR (95% CI)**	**P**	**HR (95% CI)**	**P**
**Treated hypertension (comp "healthy controls")**	1.07 (0.93, 1.23)	0.36	1.01 (0.84, 1.23)	0.88	0.99 (0.62, 1.58)	0.96
**Untreated hypertension (comp "healthy controls")**	1.37 (1.07, 1.76)	**0.012**	1.4 (1.02, 1.93)	**0.035**	1.84 (0.96, 3.53)	0.067
**Untreated hypertension (comp Treated hypertension)**	1.29 (1.02, 1.62)	**0.031**	1.39 (1.04, 1.85)	**0.026**	1.85 (1.06, 3.22)	**0.031**

Summary of Cox Proportional Hazards Models examining the effect of HT/AHT status restricted to older age groups (≥65 yo, ≥75 yo, ≥80 yo). The models were adjusted for Age, Age^2, Sex, Education, Racial group and a random intercept for Study. There were 14 studies included in the main analysis for HT/AHT status (CLAS, EAS, EPIDEMCA, ESPRIT,H70, HELIAD, Invece.Ab, KLOSCAD, PATH, SALSA, SLASI, SPAH, SydneyMAS and ZARADEMP).

© 2023 Lennon MJ et al. *JAMA Network Open.*

eTable 13. Interactions Between SBP/DBP and Age, Sex, and Ethnicity Groups

	SBP (n = 27,508, nevent = 1,668)			DBP (n = 27,499, nevent = 1,674)		
	HR (95% CI)	P	P for interaction	HR (95% CI)	P	
Age Interaction						
**Age**	1.70 (1.12, 2.59)	**0.01**		1.23 (0.89, 1.70)	0.21	
**ns(BP, 3), 1**	0.91 (0.63, 1.30)	0.60		0.68 (0.50, 0.92)	**0.01**	
**ns(BP, 3), 2**	0.73 (0.17, 3.01)	0.67		0.48 (0.15, 1.56)	0.22	
**ns(BP, 3), 3**	1.06 (0.65, 1.75)	0.81		1.07 (0.67, 1.71)	0.77	
**ns(BP, 3), 1*Age**	0.98 (0.79, 1.22)	0.88	0.29	1.20 (1.01, 1.43)	**0.04**	0.08
**ns(BP, 3), 2*Age**	1.17 (0.49, 2.81)	0.72		2.39 (1.20, 4.79)	**0.01**	
**ns(BP, 3), 3*Age**	0.91 (0.69, 1.21)	0.53		1.24 (0.95, 1.63)	0.11	
Sex Interaction						
**Sex (Female)**	**1**	-		**1**	-	
**Sex (Male)**	1.71 (0.47, 6.27)	0.42		1.25 (0.47, 3.30)	0.66	
**ns(BP, 3), 1**	1.00 (0.66, 1.51)	0.99		0.90 (0.65, 1.24)	0.50	
**ns(BP, 3), 2**	1.14 (0.21, 6.03)	0.88		1.33 (0.39, 4.51)	0.65	
**ns(BP, 3), 3**	1.01 (0.61, 1.70)	0.96		1.30 (0.80, 2.11)	0.30	
**ns(BP, 3), 1*Sex (Male)**	0.77 (0.39, 1.49)	0.43	0.88	0.79 (0.46, 1.36)	0.40	0.80
**ns(BP, 3), 2*Sex (Male)**	0.36 (0.02, 5.53)	0.46		0.78 (0.10, 6.41)	0.82	
**ns(BP, 3), 3*Sex (Male)**	0.79 (0.32, 1.92)	0.60		1.16 (0.51, 2.62)	0.73	
Ethnicity Interaction						
**Ethnicity (Asian)**	0.68 (0.11, 4.36)	0.69		2.03 (0.39, 10.68)	0.40	
**Ethnicity (Black)**	1.59 (0.25, 10.19)	0.62		1.00 (0.21, 4.67)	0.99	
**ns(BP, 3), 1**	1.02 (0.64, 1.62)	0.93		0.89 (0.65, 1.23)	0.48	
**ns(BP, 3), 2**	0.72 (0.11, 4.65)	0.73		1.23 (0.36, 4.17)	0.74	
**ns(BP, 3), 3**	0.83 (0.47, 1.46)	0.52		1.31 (0.81, 2.11)	0.27	
**ns(BP, 3), 1*Ethnicity (Asian)**	0.59 (0.26, 1.38)	0.23	0.57	0.72 (0.35, 1.47)	0.37	0.50
**ns(BP, 3), 1*Ethnicity (Black)**	0.83 (0.32, 2.14)	0.70		1.08 (0.49, 2.38)	0.85	
**ns(BP, 3), 2*Ethnicity (Asian)**	2.30 (0.07, 76.68)	0.64		0.11 (0.01, 2.22)	0.15	
**ns(BP, 3), 2*Ethnicity (Black)**	1.51 (0.04, 56.06)	0.82		3.86 (0.20, 75.90)	0.37	
**ns(BP, 3), 3*Ethnicity (Asian)**	1.71 (0.18, 16.00)	0.64		0.22 (0.03, 1.96)	0.18	
**ns(BP, 3), 3*Ethnicity (Black)**	1.61 (0.57, 4.51)	0.36		1.80 (0.69, 4.69)	0.23	

Summary of Cox Proportional Hazards Models examining the interaction between baseline SBP/DBP and Age, Sex and Ethnicity. The models were adjusted for Age, Age^2, Sex, Education and Ethnicity. There were 17 studies included in the main analysis for SBP and DBP (CLAS, EAS, EPIDEMCA, ESPRIT, H70, HELIAD, Indi_ibadan, Invece.Ab, ISA, KLOSCAD, LEILA75+, PATH, SALSA, SLASI, SPAH, SydneyMAS and ZARADEMP).

© 2023 Lennon MJ et al. *JAMA Network Open.*

eTable 14. Interaction Terms Between HT/AHT Status and BP

Interactions between HT/AHT (n = 21,737, nevent = 2,173)						
			SBP			DBP
	HR (95% CI)	P	P interact	HR (95% CI)	P	P interact
Treated Hypertension (comp "Healthy Controls")	1.38 (0.25, 7.62)	0.71		1.04 (0.29, 3.80)	0.95	
Untreated Hypertension (comp "Healthy Controls")	4.03 (0.35, 46.79)	0.26		2.87 (0.30, 27.16)	0.36	
Untreated Hypertension (comp Treated Hypertension)	2.92 (0.24, 36.20)	0.40		2.75 (0.32, 23.42)	0.36	
ns(BP, 3) 1	0.90 (0.50, 1.64)	0.73		0.81 (0.46, 1.42)	0.46	
ns(BP, 3) 2	0.90 (0.08, 10.55)	0.93		1.42 (0.15, 13.49)	0.76	
ns(BP, 3) 3	0.89 (0.31, 2.57)	0.84		1.63 (0.65, 4.11)	0.30	
Treated Hypertension (comp "Healthy Controls")* ns(BP, 3) 1	0.86 (0.36, 2.05)	0.72	0.90	1.23 (0.61, 2.50)	0.57	0.91
Untreated Hypertension (comp "Healthy Controls")* ns(BP, 3) 1	0.77 (0.19, 3.01)	0.70		0.69 (0.12, 2.39)	0.55	
Untreated Hypertension (comp Treated Hypertension)* ns(BP, 3) 1	0.90 (0.23, 3.56)	0.87		0.56 (0.17, 1.86)	0.34	
Treated Hypertension (comp "Healthy Controls")* ns(BP, 3) 2	0.63 (0.02, 23.77)	0.80		0.91 (0.06, 15.26)	0.95	
Untreated Hypertension (comp "Healthy Controls")* ns(BP, 3) 2	0.12 (0.01, 21.32)	0.42		0.19 (0.00, 23.70)	0.50	
Untreated Hypertension (comp Treated Hypertension)* ns(BP, 3) 2	0.19 (0.01, 37.01)	0.54		0.21 (0.00, 20.85)	0.51	
Treated Hypertension (comp "Healthy Controls")* ns(BP, 3) 3	0.75 (0.19, 2.91)	0.68		0.73 (0.22, 2.47)	0.61	
Untreated Hypertension (comp "Healthy Controls")* ns(BP, 3) 3	0.85 (0.15, 4.89)	0.85		0.38 (0.06, 2.40)	0.30	
Untreated Hypertension (comp Treated Hypertension)* ns(BP, 3) 3	1.12 (0.21, 5.88)	0.89		0.52 (0.09, 3.09)	0.47	

Summary of Cox Proportional Hazards Models examining the interaction between baseline SBP/DBP and HT/AHT Status. The models were adjusted for Age, Age^2, Sex, Education and Ethnicity. There were 14 studies included this analysis (CLAS, EAS, EPIDEMCA, ESPRIT, H70, HELIAD, Invece.Ab, KLOSCAD, PATH, SALSA, SLASI, SPAH, SydneyMAS and ZARADEMP).

© 2023 Lennon MJ et al. *JAMA Network Open.*

eTable 15. HT/AHT Status and Dementia, Including Those With BP >160/100 mm Hg in the Untreated Hypertension Group

	**Main analysis (BP >/= 160/100 classified as "hypertensive" irrespective of history) (n = 19,276, nevent = 1,101)**	
	**HR (95% CI)**	**P**
**Treated hypertension (comp "healthy controls")**	1.1 (0.95, 1.27)	0.18
**Untreated hypertension (comp "healthy controls")**	1.26 (1.04, 1.53)	**0.019**
**Untreated hypertension (comp Treated hypertension)**	1.14 (0.96, 1.36)	0.14

Summary of Cox Proportional Hazards Models examining relationship between HT/AHT status and dementia. In this altered analysis we categorised those not taking an antihypertensive and reporting no history of hypertension but with an SBP ≥ 160 mmHg or DBP ≥ 100 into the "Untreated hypertension" group. By contrast, our main analysis did not consider baseline blood pressure in the categorisation of hypertension.

© 2023 Lennon MJ et al. *JAMA Network Open.*
