# Supplementary material for: Use of Antihypertensives, Blood Pressure, and Estimated Risk of Dementia in Late Life: An Individual Participant Data Meta-Analysis
Source: JAMA Netw Open. 2023 Sep 12;6(9):e2333353. doi: 10.1001/jamanetworkopen.2023.33353 (PMC10498335; doi:10.1001/jamanetworkopen.2023.33353)
Supplement: Supplement 2. — Data Sharing Statement [file jamanetwopen-e2333353-s002.pdf]

Data Sharing Statement

Lennon. Use of Antihypertensives, Blood Pressure, and Estimated Risk of Dementia in Late Life. *JAMA Netw Open*. Published September 12, 2023.
doi:10.1001/jamanetworkopen.2023.33353

Data
Data available: Yes
Data types: Deidentified participant data
How to access data: Individual participant data cannot be made publicly available because they are protected by a confidentiality agreement. Data were provided by the contributing studies to COSMIC on the understanding and proviso that the relevant study leaders be contacted for further use of their data and additional formal data sharing agreements be made. Researchers can apply to use COSMIC data by completing a COSMIC Research Proposal Form available from https://cheba.unsw.edu.au/ consortia/cosmic/research-proposals.
When available: With publication

Supporting Documents
Document types: None

Additional Information
Who can access the data: Researchers whose proposed use of the data has been approved
Types of analyses: For any purpose
Mechanisms of data availability: After approval of a proposal with a signed data access agreement
